# Nuclear Small Dystrophin Isoforms during Muscle Differentiation

**DOI:** 10.3390/life13061367

**Published:** 2023-06-11

**Authors:** Tina Donandt, Vanessa Todorow, Stefan Hintze, Alexandra Graupner, Benedikt Schoser, Maggie C. Walter, Peter Meinke

**Affiliations:** Friedrich-Baur-Institute at the Department of Neurology, LMU University Hospital, Ludwig Maximilians University, 81377 Munich, Germany; tina.donandt@med.uni-muenchen.de (T.D.); vanessa.todorow@med.uni-muenchen.de (V.T.); stefan.hintze@med.uni-muenchen.de (S.H.); alexandra.graupner@med.uni-muenchen.de (A.G.); benedikt.schoser@med.uni-muenchen.de (B.S.); maggie.walter@med.uni-muenchen.de (M.C.W.)

**Keywords:** Duchenne muscular dystrophy, muscle cell culture, DMD, dystrophin, porcine muscle cells, nuclear localization

## Abstract

Mutations in the *DMD* gene can cause Duchenne or Becker muscular dystrophy (DMD/BMD) by affecting the giant isoform of dystrophin, a protein encoded by the *DMD* gene. The role of small dystrophin isoforms is not well investigated yet, and they may play a role in muscle development and molecular pathology. Here, we investigated the nuclear localization of short carboxy-terminal dystrophin isoforms during the in vitro differentiation of human, porcine, and murine myoblast cultures. We could not only confirm the presence of Dp71 in the nucleoplasm and at the nuclear envelope, but we could also identify the Dp40 isoform in muscle nuclei. The localization of both isoforms over the first six days of differentiation was similar between human and porcine myoblasts, but murine myoblasts behaved differently. This highlights the importance of the porcine model in investigating DMD. We could also detect a wave-like pattern of nuclear presence of both Dp71 and Dp40, indicating a direct or indirect involvement in gene expression control during muscle differentiation.

## 1. Introduction

The *DMD* gene (ENSG00000198947; Xp21.2-p21.1) gives rise to multiple dystrophin isoforms, which are expressed in a tissue-specific manner. The usage of different promoters, alternative splicing, and alternative poly-A addition sites are factors involved in generating the multiple isoforms described [1,2,3]. The resulting dystrophin proteins (Dp) have been named according to their molecular mass. So far, the described proteins include Dp427 (full-length dystrophin), Dp260, Dp140, Dp116, Dp71, and Dp40, which are found in different tissues, such as the brain, muscle, retina, kidney, peripheral nerves, liver and lung [1,4].

Dp427 is crucial for the strength and stability of muscle fibers, and its absence results in fiber degeneration [5]. The loss of full-length dystrophin results in Duchenne muscular dystrophy (DMD) [6], while a partial loss, leading to a shortened protein with a reduced function, results in clinically milder Becker muscular dystrophy (BMD) [7]. The role of other dystrophin isoforms in DMD/BMD is implicated but not yet completely solved [8,9,10,11,12].

The carboxy-terminal isoform Dp71, which is expressed ubiquitously, has been linked to myoblast proliferation [13] and neural stem/progenitor cell proliferation and differentiation [14]. This isoform has been shown to localize within and closely around the nucleus of PC12, HeLa, C2C12 and HEK293 cells and human myoblasts [13,15,16,17,18,19], but also to mitochondria [20]. Depletion of Dp71 alters the distribution of aquaporin-4 channels in brain macroglial cells, but the question of which function Dp71 fulfils in the nucleus and how stable it appears there remains unclear.

Another short dystrophin isoform, Dp40, is expressed from the same promoter as Dp71. Dp40 localizes partially to the nucleus in PC12 cells [21,22], appears to be essential for PC12 neuronal differentiation [23] and has a crucial role in presynaptic function [24].

Here, we studied in vitro the localization and expression of carboxy-terminal dystrophin isoforms in differentiating myotubes over the first six days of differentiation in human, porcine and murine myoblast cultures.

## 2. Materials and Methods

### 2.1. Tissue Culture

Primary human myoblasts were obtained from the Muscle Tissue Culture Collection (MTCC) at the Friedrich-Baur-Institute (Department of Neurology, LMU Klinikum, Ludwig-Maximilians-University, Munich, Germany). The human myoblast culture was obtained with written informed consent of the donor. Ethical approval for this study was obtained from the ethical review committee at the Ludwig-Maximilians-University, Munich, Germany (reference 45-14). Primary porcine myoblasts were obtained as described elsewhere [25]. Mouse myoblasts were kindly provided by Nina Raben.

Human myoblasts were cultured in Skeletal Muscle Growth Medium (PELOBiotech, Munich, Germany) supplemented with GlutaMax, 40 U/mL Penicillin, 0.04 mg/mL Streptomycin, and SkMC supplement. The medium was changed to DMEM containing 5% horse serum for differentiation. Cells were kept in an incubator at 37 °C and 5% CO_2_.

Porcine myoblasts were grown in α-MEM supplemented with 10% FCS, 1% Glutamax, 40 U/mL Penicillin, and 0.04 mg/mL Streptomycin on plates coated with Fibronectin (Sigma Aldrich #F0895). For differentiation, the medium was switched to DMEM high glucose with 0.4% Ultroser G. Cells were kept in an incubator at 37 °C and 5% CO_2_.

Mouse myoblasts were cultured in high-glucose DMEM supplemented with 10% FCS, 40 U/mL penicillin, and 0.04 mg/mL Streptomycin at 33 °C with 5% CO_2_. To start differentiation, the medium was switched to high-glucose DMEM supplemented with 5% horse serum, 40 U/mL penicillin, and 0.04 mg/mL Streptomycin. Cells were incubated at 37 °C with 5% CO_2_ for differentiation.

During the last passaging before differentiation, several coverslips were placed in the culture dishes. Myoblasts were grown until they were close to reaching confluence. Differentiation medium was exchanged every two days, and cells were differentiated over six days.

### 2.2. Immunofluorescence Staining

From the starting point of differentiation (day 0) until the last day (day 6), coverslips were fixed with ice-cold methanol on each day. Cells were stained for dystrophin (abcam ab 15277, 1:50) and DAPI. The secondary antibody used was Alexa Four Plus 488 (Invitrogen, Waltham, MA, USA: A32790, anti-rabbit, 1:500). Immunofluorescence pictures were taken with an Olympus IX83 microscope and U Plan X Apo 40x objective. All experiments were repeated at least three times.

### 2.3. Western Blot

Cell pellets of every differentiation day were resuspended in 1 mL of hypotonic lysis buffer (10 mM HEPES pH 7.4, 1.5 mM MgCl_2_, 2 nM DTT) and could swell for 10–15 min on ice. Nuclei were released by Dounce homogenization of 20 vigorous strokes using a type B pestle (Wheaton, clearance between 0.1 and 0.15 mm). Thereafter, 100 µL of 1 M KCl were added. Nuclei were then pelleted at 2000× *g* in a swinging bucket rotor for 20 min at 4 °C. The supernatant was transferred into a new 1.5 mL Eppendorf tube, and the pellet was resuspended in 1 mL PBS and pelleted again at 2000× *g* in a swinging bucket rotor for 20 min at 4 °C. The second supernatant was discarded. The nuclei pellets were dissolved in 80 µL RIPA buffer (50 mM Tris/HCl pH 8.0, 150 mM NaCl 1% NP40 0.5% sodium deoxycholate, 0.1% SDS, freshly added protease inhibitor tablet from Roche (Complete Ultra protease inhibitors #05 892 970 001)) and then ultrasonicated with a Bandelin Sonopuls ultrasonicator (two times 15 s, 5 × 10%).

Western blot was performed using BioRad’s Tetra electrophoresis chamber and the TransBlot Turbo^®^ system for protein blotting (BioRad, Hercules, CA, USA). A total of 20 µg whole protein was used per lane in the different blots. An antibody against lamin A/C (4C10, provided by Glenn E. Morris, 1:500) was used to control the presence of nuclei. Dystrophin (Abcam, Cambridge, UK, ab15266, 1:500 or 1:1000 in 3% BSA/TBST) was used as primary antibody. Desmin (Novus Biological, Littleton, CO, USA, NBP1-45143, 1:1000) was used to control the separation of nuclei. A control for the consistent protein loading of all lanes Total Protein Stain (LiCor, Lincoln, NE, USA) was used. All Western blots were detected in the LiCor Odyssey Fc Imaging System using fluorescent secondary antibodies (Donkey anti-rabbit 800 CW 926-32213, Donkey anti-mouse 680RD 926-68072). All experiments were repeated at least three times.

### 2.4. RNA-Sequencing

Human myoblasts were harvested at 50% confluence. During the differentiation, cells were harvested at day 0 (90% confluence) and then at days 1, 2, 4, and 6. After two washing steps with 1× PBS, Trizol^®^ was added to the samples. The Trizol^®^/sample mixture was used directly to isolate the RNA using the Dircet-zol^TM^ RNA MiniPrep PlusKit (ZYMO Research, Freiburg, Germany). Library preparation was performed using the TruSeq Stranded mRNA Kit (Illumina, San Diego, CA, USA) with the TruSeq RNA Single Index Set A (Illumina) according to the recommended procedure. The quality and size distribution of the generated libraries has been validated using an Agilent 2100 bioanalyzer with a high-sensitivity DNA chip (Agilent, Santa Clara, CA, USA). DNA yield was measured using the Qubit dsDNA HS Assay Kit, followed by pooling of the libraries in batches of 12 and sequencing of 1.2 pM pooled library on an Nextseq 500/High Output Flow Cell Cartridge using a paired-end, 2 × 76 reads, single-index protocol. Experiments were performed in triplicates.

A sufficient quality of the RNAseq data from was confirmed using FASTQC. Raw reads were mapped to the human GRCh38 genome using STAR v2.7 and sorted with the in-built SortedByCoordinate function, while index files were generated with samtools. The resulting BAM and index files were loaded into the Integrative Genomics Viewer (IGV) web application. Reads mapping to specific exons were counted and normalized against the total number of reads per sample.

### 2.5. Statistical Analysis

Differences between the different timepoints of differentiation (per dystrophin isoform and per species) were calculated by a non-parametric test (Kruskal-Wallis-Test). The corresponding *p*-values as well as a statistical comparison of any pair of groups are included in Supplementary Figure S3.

## 3. Results

### 3.1. Localization of C-Terminal Dys during Muscle Differentiation

**Figure 1:**
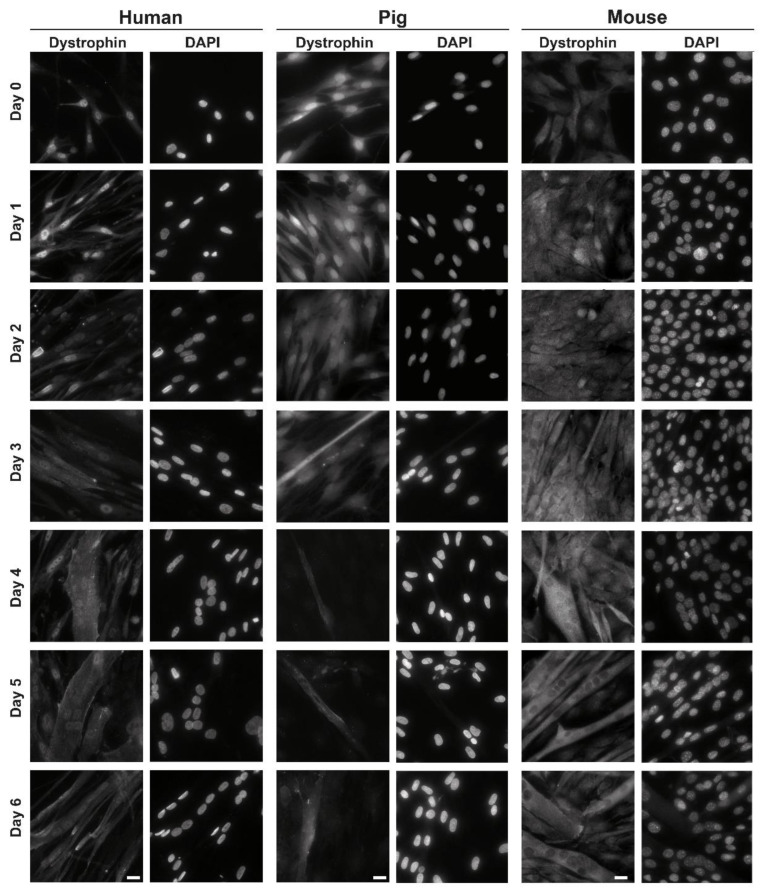
Human (left), porcine (middle) and murine (right) myoblast cultures were differentiated over 6 days. While changing to differentiating medium at day 0 and then every 24 h, coverslips were harvested, fixed and stained with dystrophin (left side) and DAPI (right side). Scale bar, 20 µm.

We stained dystrophin in human, porcine, and murine differentiating myoblast cultures from day 0 to 6 of differentiation to study the development of dystrophin isoform localization during differentiation. In all species and during all time points, we could detect immunofluorescent staining of dystrophin (Figure 1).

In human myoblasts, there was dystrophin staining within the nucleus and in the cytoplasm at all observer time points. There were, however, differences in the intensity of the staining at the respective cellular location. In proliferating myoblasts (day 0) and until day 2 of differentiation, the nucleoplasmic dystrophin staining was more intense than the cytoplasmic one. At all days, there was also the appearance of nuclear envelope staining in some nuclei (Supplementary Figure S1). From day 3 on, cytoplasmic staining became more intense, especially in forming myotubes (Figure 1, left panel).

In porcine myoblast cultures, there was—similar to human myoblasts—more intensive nucleoplasmic staining from day 0 to day 2 of differentiation. We could also observe a clear nuclear rim staining at these time points. From day 3 to day 6, there was dystrophin staining in the cytoplasm and nucleoplasm of myotubes. In single-nucleated cells, dots within the nuclei were stained (Figure 1, middle panel).

In murine myoblast cultures, we also observed dystrophin staining at all days of differentiation. From day 0 to day 2, there was roughly equal cytoplasmic and nucleoplasmic staining, but from day 3 on, in myotubes, we observed only cytoplasmic staining. At the same time, there was no signal within the nuclei (Figure 1, right panel).

Experiments were repeated at least three times, and occasional nuclear-envelope staining was observed in human as well as porcine cultures up to day 5 of differentiation (in myotubes) and in murine myoblast cultures up to day 2 of differentiation (single nucleated cells; Supplementary Figure S1).

### 3.2. Quantification of C-Terminal Dystrophin during Muscle Differentiation and RNAseq of Human Myoblast Differentiation

To identify the nuclear dystrophin isoform/isoforms, we isolated nuclei of human, porcine, and murine differentiating myoblast cultures from day 0 to day 6 of differentiation. Separated nuclei were probed via Western blot for the presence of dystrophin isoforms during differentiation.

**Figure 2:**
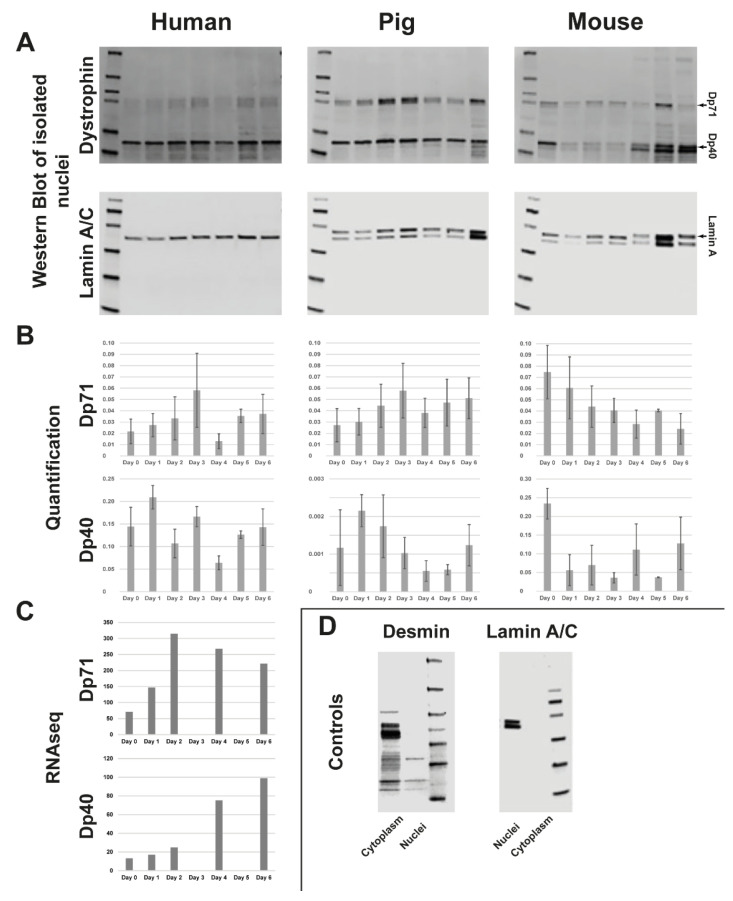
(**A**) Western blot of separated nuclei from human (left), porcine (middle) and murine (right) myotubes during differentiation from days 0–6. Progression of dystrophin signal in Dp 71 and Dp 40 from day 0 (left side) to day 6 (right side). Each Western blot used the nuclear-envelope protein lamin A as a loading control. (**B**) Dystrophin signal (Dp 71 and Dp 40) from each species from days 0–6 of differentiation was quantified. (**C**) RNAseq of human myoblasts during differentiation from days 0–6. (**D**) Samples were tested for the successful separation of nuclei from cytoplasm with cytoplasmic protein desmin and nuclear-envelope protein lamin A/C (example shown: murine myoblasts). Full pictures of all Western blots are presented in Supplementary Figure S2.

In human myoblasts, we identified a dystrophin signal probably originating from two bands slightly bigger than 70 kDa at all days of differentiation. The size of these bands fits with Dp71, for which several variants (DP71a, Dp71b, and DP71ab) have been described. There was also a strong signal at about 32 kDa (running below 40 kDa, as also shown elsewhere [21]) and a ladder of weaker smaller bands at all days (Figure 2A, left panel). We also obtained a clear signal for lamin A at all timepoints, which was used to normalize the dystrophin isoforms. Quantification of the DP71 and DP40 bands showed a wave-like picture for DP71, with a constantly increasing signal between day 0 and day 3, a drop on day 4, and an increase until day 6. The DP40 signal also provided a wave-like picture, with an increase from day 0 to day 1, a constant drop until day 4, and an increase from thereon until day 6. Only the day 3 signal deviated by being higher than the trend (Figure 2B, left panel).

As the actual size of the band we identified as Dp40 differs from the expected size (~32 kDa instead of 40 kDa), we utilized RNA-sequencing data of human myoblast differentiation (days 0, 1, 2, 4, 6) to investigate the expression of the exons contributing to the respective isoforms. Dp40 shares all exons with Dp427, and Dp71 and Dp71 share all except one exon with Dp427. This results in the following difference: amino acids KVPYYIN (aa 3069 to 3075 in Dp427) are exchanged to MREQLKG (aa 1 to 7 in Dp71). First, we used this difference to determine the difference between Dp71 and Dp472 expression. As the exons giving rise to Dp40 are shared with Dp71, we then subtracted the average number of reads for Dp472 and Dp71 from the reads in the exons encoding all three isoforms, thus obtaining the average reads for Dp40. This analysis did show a peak of Dp71 expression at day 2, while Dp40 expression constantly increased until day 6 (Figure 2C).

In porcine myoblast cultures, there were also dystrophin signals of the same size as Dp71 and Dp40; overall, the picture was similar to human myoblasts. The lamin A/C antibody stained both lamin A and lamin C (Figure 2A, middle panel). We used lamin a for normalization, and the quantification did show—similar to human myoblasts—a peak of Dp71 in the nucleus at 3 days of differentiation, followed by a drop and increase of the signal until day 6. Dp40 did, also similar to human myoblasts, show a peak of nuclear localization at day 2 followed by a drop until day 4 and an increase from day 4 to day 6 (Figure 2B, middle panel).

In murine myoblasts, all the dystrophin bands observed in human and porcine myoblasts were present as well. The only difference was a more intense band below the Dp40 band from day 4 (Figure 2A, right panel). Quantification of nuclear dystrophin did, however, reveal a different picture than that observed in human and porcine myoblasts. The nuclear localization of Dp71 was strongest in proliferating myoblasts and dropped off constantly during differentiation. Dp40 also had the strongest nuclear localization in proliferating cells, with lower but varying amounts in the following days (Figure 2B, right panel).

To ensure that the separation of the nuclei worked without contamination of the respective other fraction, the nuclear and cytoplasmic fractions were both tested for lamin A/C as a nuclear marker and desmin as a cytoplasmic marker by Western blot. This showed that lamin A/C was only present in the nuclear fraction and desmin (~53 kDa) only in the cytoplasmic fraction (examples shown in Figure 2D).

## 4. Discussion

Mutations in the *DMD* gene are causing DMD, BMD, and dilated cardiomyopathy [26,27]. They can be located all over the *DMD* gene [28], thus potentially affecting different dystrophin isoforms [1]. While the role of full-length dystrophin in the disease pathology is well investigated, the contribution of small isoforms still needs to be solved. The isoform Dp71 is mainly found in brain tissue, but also in myogenic cells and skeletal muscle [8]. Recent findings indicate an important role of Dp71 in neuronal tissue [29]. This makes it a main interest in DMD research, as this is related to a non-progressive cognitive impairment in DMD [30] without always being combined with muscle weakness [31,32]. Despite this, the function of short dystrophin isoforms in muscle is still not completely solved, and its potential influence on the muscle phenotype of DMD/BMD is unknown.

After noticing varying occurrences of nuclear dystrophin staining using an antibody against the c-terminus of dystrophin, we systematically investigated the presence and localization of nuclear short dystrophin isoforms during early differentiation. We could identify a nuclear localization of Dp71 and Dp40 throughout the first six days of in vitro muscle differentiation. Dp71 and Dp40 do not have a nuclear localization signal. Instead, phosphorylation is thought to be the regulator of nuclear import [29]. It has been shown before that Dp71 enhances the proliferation of myoblasts [13] and can localize to the nucleus and to the nuclear envelope, where it stabilizes nuclear dystrophin-associated proteins [15,17]. We could confirm the nuclear and nuclear-envelope localization of Dp71. However, the increasing nuclear presence until day three of differentiation (and after that again an increased nuclear presence from day four until day six) in human and porcine myoblasts indicates a function different to proliferation enhancement, as the myoblasts are not proliferative anymore from day one of differentiation. The lack of lamin C in human myoblasts may be due to epitope masking, as has been shown for the nuclear envelope proteins lamin B1 and emerin before [33].

The nuclear localization of Dp40 has been shown in PC12 cells [22], and here we can additionally prove it for muscle cells. As the actual Western blot band was running lower than 40 kDa, we confirmed the expression of Dp40 using RNAseq data. During muscle differentiation, the nuclear presence of both human and porcine Dp71 and Dp40 appears to be in waves and is shifted against one other. This may indicate a distinct function in gene expression control, which is undergoing major alterations during muscle differentiation. Expression waves have been described for myogenesis [34], although not in the context of dystrophin.

Another critical question is which model system should be used to investigate the role of short dystrophin isoforms in muscle differentiation and disease. For DMD, the DMD pig is a superior animal model closely mirroring the human phenotype [35], and there is a cell culture system available [25]. The results presented here highlight the importance of the porcine model system. The porcine myoblasts show a nuclear localization of short dystrophin isoforms very similar to human myoblasts during early differentiation. Murine myoblasts did deviate regarding this nuclear localization, but it must be noted that the *mdx* mouse also displays a very mild phenotype compared to humans or pigs [36,37].

In summary, we can extend the knowledge about short dystrophin isoforms during muscle differentiation. Furthermore, it will be helpful to advance research on the function of Dp71 and Dp40 during differentiation to understand the molecular function and the relevance of human disease.

## Data Availability

The data presented in this study are available in this article (and supplementary material).

## Supplementary Materials

The following supporting information can be downloaded at: https://www.mdpi.com/article/10.3390/life13061367/s1, Figure S1: Examples of nuclear envelope dystrophin staining; Figure S2: All Western blots used for quantification; Figure S3: Statistical analysis of the nuclear presence of both Dp71 and Dp40 for the seven timepoints analyzed per species.
