# Supplementary material for: Nuclear Small Dystrophin Isoforms during Muscle Differentiation
Source: Life (Basel). 2023 Jun 11;13(6):1367. doi: 10.3390/life13061367 (PMC10302385; doi:10.3390/life13061367)
Supplement: Supplementary file 1 [file life-13-01367-s001.zip › Supplemental figure S3.pdf]

DP71 human *P* = 0.0633

P-value	Day 0	Day 1	Day 2	Day 3	Day 4	Day 5	Day 6
**Day 0**		0.6673	0.4391	0.0352	0.3667	0.1561	0.1973
**Day 1**	0.6673		0.7310	0.0937	0.1827	0.3229	0.3900
**Day 2**	0.4391	0.7310		0.1827	0.0937	0.5191	0.6060
**Day 3**	0.0352	0.0937	0.1827		0.0026	0.4917	0.4141
**Day 4**	0.3667	0.1827	0.0937	0.0026		0.0203	0.0284
**Day 5**	0.1561	0.3229	0.5191	0.4917	0.0203		0.8974
**Day 6**	0.1973	0.3900	0.6060	0.4141	0.0284	0.8974	

DP71 pig *P* = 0.4791

P-value	Day 0	Day 1	Day 2	Day 3	Day 4	Day 5	Day 6
**Day 0**		0.4318	0.1511	0.0293	0.2325	0.1063	0.0609
**Day 1**	0.4318		0.1950	0.0428	0.2882	0.1413	0.0845
**Day 2**	0.1511	0.1950		0.1950	0.3818	0.4149	0.3030
**Day 3**	0.0293	0.0428	0.1950		0.1229	0.2596	0.3655
**Day 4**	0.2325	0.2882	0.3818	0.1229		0.3030	0.2071
**Day 5**	0.1063	0.1413	0.4149	0.2596	0.3030		0.3818
**Day 6**	0.0609	0.0845	0.3030	0.3655	0.2071	0.3818	

DP71 mouse *P* = 0.3167

P-value	Day 0	Day 1	Day 2	Day 3	Day 4	Day 5	Day 6
**Day 0**		0.5998	0.4068	0.2535	0.0598	0.3105	0.0387
**Day 1**	0.5998		0.7539	0.4902	0.1290	0.5576	0.0845
**Day 2**	0.4068	0.7539		0.6714	0.1904	0.7327	0.1258
**Day 3**	0.2535	0.4902	0.6714		0.4076	0.9754	0.3006
**Day 4**	0.0598	0.1290	0.1904	0.4076		0.4404	0.8360
**Day 5**	0.3105	0.5576	0.7327	0.9754	0.4404		0.3387
**Day 6**	0.0387	0.0845	0.1258	0.3006	0.8360	0.3387	

DP40 human *P* = 0.03878

P-value	Day 0	Day 1	Day 2	Day 3	Day 4	Day 5	Day 6
**Day 0**		0.1882	0.2925	0.6451	0.0564	0.5106	0.9999
**Day 1**	0.1882		0.0179	0.3924	0.0013	0.0484	0.1882
**Day 2**	0.2925	0.0179		0.1302	0.3924	0.6930	0.2925
**Day 3**	0.6451	0.3924	0.1302		0.0179	0.2633	0.6451
**Day 4**	0.0564	0.0013	0.3924	0.0179		0.2113	0.0564
**Day 5**	0.5106	0.0484	0.6930	0.2633	0.2113		0.5106
**Day 6**	0.9999	0.1882	0.2925	0.6451	0.0564	0.5106	

DP40 pig *P* = 0.1165

P-value	Day 0	Day 1	Day 2	Day 3	Day 4	Day 5	Day 6
**Day 0**		0.1478	0.3570	0.8953	0.2633	0.3570	0.7924
**Day 1**	0.1478		0.5986	0.1143	0.0103	0.0176	0.2363
**Day 2**	0.3570	0.5986		0.2925	0.0414	0.0654	0.5106
**Day 3**	0.8953	0.1143	0.2925		0.3237	0.4298	0.6930
**Day 4**	0.2633	0.0103	0.0414	0.3237		0.8435	0.1671
**Day 5**	0.3570	0.0176	0.0654	0.4298	0.8435		0.2363
**Day 6**	0.7924	0.2363	0.5106	0.6930	0.1671	0.2363	

DP40 mouse *P* = 0.1922

P-value	Day 0	Day 1	Day 2	Day 3	Day 4	Day 5	Day 6
**Day 0**		0.0281	0.0519	0.0104	0.1649	0.0630	0.2801
**Day 1**	0.0281		0.8682	0.5675	0.4066	0.9611	0.2306
**Day 2**	0.0519	0.8682		0.4902	0.5346	0.9262	0.3340
**Day 3**	0.0104	0.5675	0.4902		0.1898	0.5998	0.0977
**Day 4**	0.1649	0.4066	0.5346	0.1898		0.5169	0.7301
**Day 5**	0.0630	0.9611	0.9262	0.5998	0.5169		0.3387
**Day 6**	0.2801	0.2306	0.3340	0.0977	0.7301	0.3387	
