# Supplementary figures and images for: Nuclear Small Dystrophin Isoforms during Muscle Differentiation
Source: Life (Basel). 2023 Jun 11;13(6):1367. doi: 10.3390/life13061367 (PMC10302385; doi:10.3390/life13061367)

**DAPI****Dystrophin****Lamin A/C****Merge****Human day 1**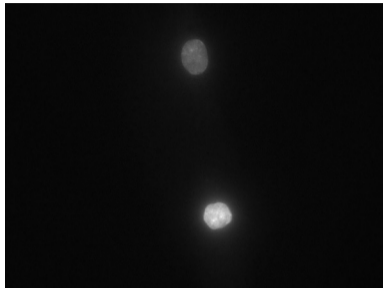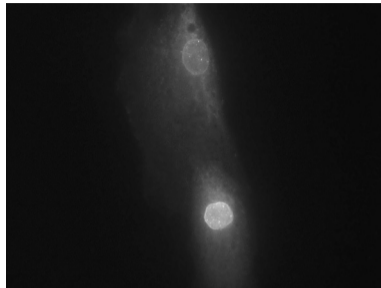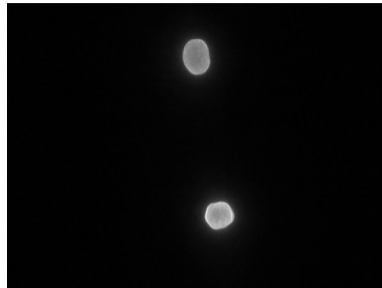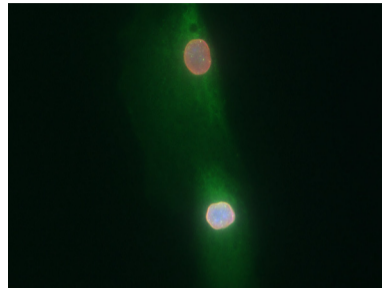**Human day 3**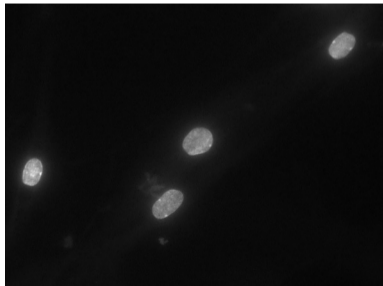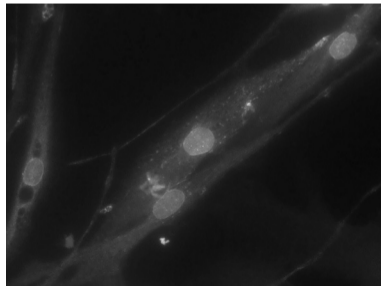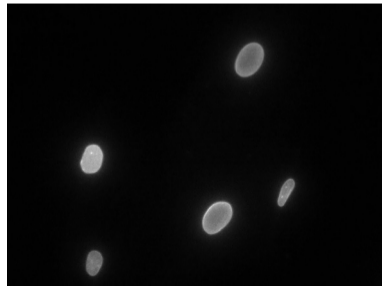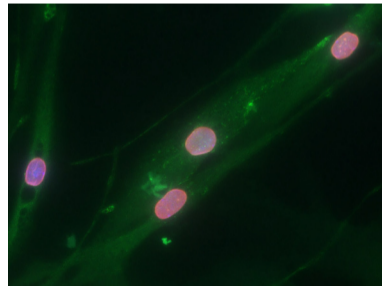

Supplement: Supplementary file 1 [file life-13-01367-s001.zip › Supplemental Figure S1.pdf]
