# Supplementary material for: Nuclear Small Dystrophin Isoforms during Muscle Differentiation
Source: Life (Basel). 2023 Jun 11;13(6):1367. doi: 10.3390/life13061367 (PMC10302385; doi:10.3390/life13061367)

Western Blot #79 Gel 1									
Image Name	Channel	Name	Signal	Total	Area	Bkgnd.	Type		
0001638_04	700	1	2.95	4.87	855	0.00225	Signal		WT H T 0
0001638_04	700	2	2.37	4.54	855	0.00254	Signal		WT H T 1
0001638_04	700	3	6.03	8.35	855	0.00271	Signal		WT H T 2
0001638_04	700	4	5.16	7.12	855	0.00229	Signal		WT H T 3
70 kDa obere Bande									
0001638_04	800	5	0.0479	0.406	770	0.000465	Signal	0.02	WT H T 0
0001638_04	800	6	0.0169	0.5	770	0.000627	Signal	0.01	WT H T 1
0001638_04	800	7	0.0935	0.622	770	0.000686	Signal	0.02	WT H T 2
0001638_04	800	8	0.0847	0.528	770	0.000576	Signal	0.02	WT H T 3
70 kDa untere Bande									
0001638_04	800	9	0.0234	0.401	770	0.000491	Signal	0.01	WT H T 0
0001638_04	800	10	0.0163	0.494	770	0.00062	Signal	0.01	WT H T 1
0001638_04	800	11	0.091	0.658	770	0.000737	Signal	0.02	WT H T 2
0001638_04	800	12	0.0963	0.564	770	0.000608	Signal	0.02	WT H T 3
30 kDa obere Bande									
0001638_04	800	13	0.501	0.989	915	0.000533	Signal	0.17	WT H T 0
0001638_04	800	14	0.414	1.01	915	0.000654	Signal	0.17	WT H T 1
0001638_04	800	15	0.652	1.21	915	0.000612	Signal	0.11	WT H T 2
0001638_04	800	16	0.729	1.28	915	0.000598	Signal	0.14	WT H T 3
30 kDa untere Bande									
0001638_04	800	17	0.0419	0.549	915	0.000554	Signal	0.01	WT H T 0
0001638_04	800	18	0.0423	0.597	915	0.000607	Signal	0.02	WT H T 1
0001638_04	800	19	0.0888	0.704	915	0.000672	Signal	0.01	WT H T 2
0001638_04	800	20	0.0627	0.69	915	0.000686	Signal	0.01	WT H T 3
260 kDa									
0001638_04	800	21	-0.000273	0.223	705	0.000316	Signal	0.00	WT H T 0
0001638_04	800	22	0.00409	0.341	705	0.000478	Signal	0.00	WT H T 1
0001638_04	800	23	0.00105	0.354	705	0.000501	Signal	0.00	WT H T 2
0001638_04	800	24	0.00555	0.295	705	0.000411	Signal	0.00	WT H T 3

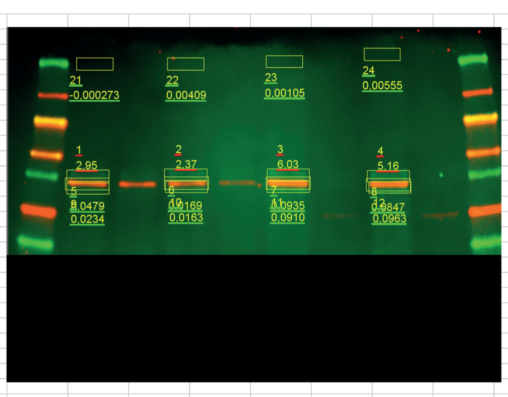

Western Blot #79 Gel 2									
Image Name	Channel	Name	Signal	Total	Area	Bkgnd.	Type		
0001639_05	700	1	7.45	10.2	855	0.00325	Signal		WT H T 4
0001639_05	700	2	11.6	14.7	855	0.00368	Signal		WT H T 5
0001639_05	700	3	7.85	10.5	855	0.00309	Signal		WT H T 6
70 kDa obere Bande									
0001639_05	800	4	0.0988	0.685	840	0.000698	Signal	0.01	WT H T 4
0001639_05	800	5	0.228	0.843	840	0.000732	Signal	0.02	WT H T 5
0001639_05	800	6	0.168	0.717	840	0.000654	Signal	0.02	WT H T 6
70 kDa untere Bande									
0001639_05	800	7	0.0583	0.665	840	0.000723	Signal	0.01	WT H T 4
0001639_05	800	8	0.221	0.899	840	0.000808	Signal	0.02	WT H T 5
0001639_05	800	9	0.178	0.786	840	0.000724	Signal	0.02	WT H T 6
30 kDa obere Bande									
0001639_05	800	10	0.547	1.37	1008	0.000819	Signal	0.07	WT H T 4
0001639_05	800	11	1.38	2.31	1008	0.000916	Signal	0.12	WT H T 5
0001639_05	800	12	1.18	1.98	1008	0.000789	Signal	0.15	WT H T 6
30 kDa untere Bande									
0001639_05	800	13	0.0756	0.934	1008	0.000851	Signal	0.01	WT H T 4
0001639_05	800	14	0.203	1.2	1008	0.000989	Signal	0.02	WT H T 5
0001639_05	800	15	0.153	1.04	1008	0.000879	Signal	0.02	WT H T 6
260 kDa									
0001639_05	800	16	0.00483	0.591	1008	0.000582	Signal	0.00	WT H T 4
0001639_05	800	17	0.00835	0.601	1008	0.000588	Signal	0.00	WT H T 5
0001639_05	800	18	0.0125	0.565	1008	0.000548	Signal	0.00	WT H T 6

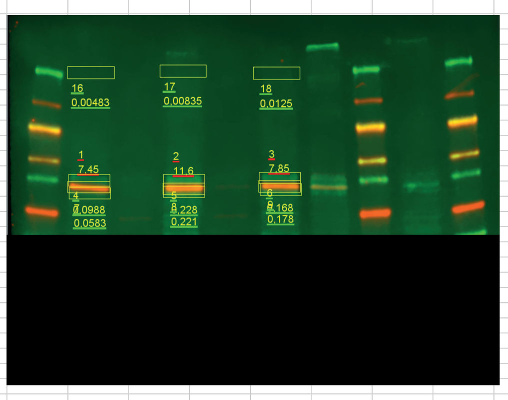

Western Blot #79 Gel 3									
Image Name	Channel	Name	Signal	Total	Area	Bkgnd.	Type		
0001640_05	700	1	2.73	4.59	689	0.00271	Signal		WT H T 0
0001640_05	700	2	2.35	4.08	689	0.0025	Signal		WT H T 1
0001640_05	700	3	4.28	6.16	689	0.00272	Signal		WT H T 2
0001640_05	700	4	2.27	4.06	689	0.00259	Signal		WT H T 3
0001640_05	700	5	4.56	6.52	689	0.00285	Signal		WT H T 4
0001640_05	700	6	6.19	8.63	689	0.00354	Signal		WT H T 5
0001640_05	700	7	4.83	6.98	689	0.00313	Signal		WT H T 6
70 kDa obere Bande									
0001640_05	800	8	0.0626	0.736	864	0.000779	Signal	0.02	WT H T 0
0001640_05	800	9	0.0686	0.722	864	0.000756	Signal	0.03	WT H T 1
0001640_05	800	10	0.159	0.805	864	0.000748	Signal	0.04	WT H T 2
0001640_05	800	11	0.134	0.806	864	0.000778	Signal	0.06	WT H T 3
0001640_05	800	12	0.0349	0.728	864	0.000802	Signal	0.01	WT H T 4
0001640_05	800	13	0.165	0.878	864	0.000825	Signal	0.03	WT H T 5
0001640_05	800	14	0.124	0.866	864	0.000859	Signal	0.03	WT H T 6
70 kDa untere Bande									
0001640_05	800	15	0.0421	0.729	864	0.000795	Signal	0.02	WT H T 0
0001640_05	800	16	0.0305	0.704	864	0.00078	Signal	0.01	WT H T 1
0001640_05	800	17	0.118	0.809	864	0.0008	Signal	0.03	WT H T 2
0001640_05	800	18	0.126	0.836	864	0.000822	Signal	0.06	WT H T 3
0001640_05	800	19	0.0463	0.743	864	0.000806	Signal	0.01	WT H T 4
0001640_05	800	20	0.0988	0.878	864	0.000902	Signal	0.02	WT H T 5
0001640_05	800	21	0.174	0.916	864	0.000859	Signal	0.04	WT H T 6
30 kDa obere Bande									
0001640_05	800	22	0.0655	0.722	784	0.000838	Signal	0.02	WT H T 0
0001640_05	800	23	0.0859	0.734	784	0.000827	Signal	0.04	WT H T 1
0001640_05	800	24	0.129	0.785	784	0.000837	Signal	0.03	WT H T 2
0001640_05	800	25	0.147	0.867	784	0.000918	Signal	0.06	WT H T 3
0001640_05	800	26	0.0857	0.829	784	0.000948	Signal	0.02	WT H T 4
0001640_05	800	27	0.281	1.03	784	0.000954	Signal	0.05	WT H T 5
0001640_05	800	28	0.275	0.993	784	0.000915	Signal	0.06	WT H T 6
30 kDa untere Bande									
0001640_05	800	29	0.0327	0.659	784	0.000799	Signal	0.01	WT H T 0
0001640_05	800	30	0.0255	0.717	784	0.000881	Signal	0.01	WT H T 1
0001640_05	800	31	0.0312	0.833	784	0.00102	Signal	0.01	WT H T 2
0001640_05	800	32	0.0604	0.82	784	0.000969	Signal	0.03	WT H T 3
0001640_05	800	33	0.051	0.836	784	0.001	Signal	0.01	WT H T 4
0001640_05	800	34	0.138	0.958	784	0.00105	Signal	0.02	WT H T 5
0001640_05	800	35	0.0711	0.795	784	0.000923	Signal	0.01	WT H T 6
260 kDa									
0001640_05	800	36	-0.00131	0.631	936	0.000676	Signal	0.00	WT H T 0
0001640_05	800	37	0.00153	0.642	936	0.000685	Signal	0.00	WT H T 1
0001640_05	800	38	0.00393	0.648	936	0.000688	Signal	0.00	WT H T 2
0001640_05	800	39	0.00545	0.64	936	0.000678	Signal	0.00	WT H T 3
0001640_05	800	40	0.00179	0.617	936	0.000657	Signal	0.00	WT H T 4
0001640_05	800	41	0.00632	0.624	936	0.00066	Signal	0.00	WT H T 5
0001640_05	800	42	0.00639	0.641	936	0.000678	Signal	0.00	WT H T 6

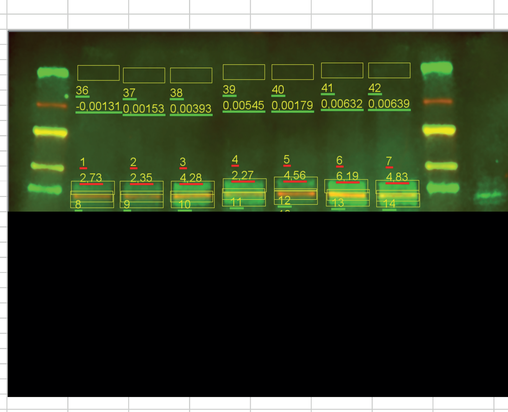

Western Blot #80 Gel 2									
Image Name	Channel	Name	Signal	Total	Area	Bkgnd.	Type		
0001646_05	700	1	4.27	5.92	848	0.00195	Signal		WT H T 0
0001646_05	700	2	3.69	5.22	848	0.00181	Signal		WT H T 1
0001646_05	700	3	6.06	7.73	848	0.00197	Signal		WT H T 2
0001646_05	700	4	6.31	8.01	848	0.002	Signal		WT H T 3
0001646_05	700	5	6.39	8.11	848	0.00203	Signal		WT H T 4
0001646_05	700	6	10.4	12.5	848	0.00249	Signal		WT H T 5
0001646_05	700	7	7	8.97	848	0.00233	Signal		WT H T 6
70 kDa obere Bande									
0001646_05	800	8	0.0615	0.799	936	0.000788	Signal	0.01	WT H T 0
0001646_05	800	9	0.0786	0.804	936	0.000775	Signal	0.02	WT H T 1
0001646_05	800	10	0.112	0.856	936	0.000795	Signal	0.02	WT H T 2
0001646_05	800	11	0.159	0.874	936	0.000763	Signal	0.03	WT H T 3
0001646_05	800	12	0.0341	0.763	936	0.000779	Signal	0.01	WT H T 4
0001646_05	800	13	0.16	0.91	936	0.000801	Signal	0.02	WT H T 5
0001646_05	800	14	0.116	0.898	936	0.000835	Signal	0.02	WT H T 6
70 kDa untere Bande									
0001646_05	800	15	0.00101	0.809	936	0.000863	Signal	0.00	WT H T 0
0001646_05	800	16	0.00584	0.819	936	0.000868	Signal	0.00	WT H T 1
0001646_05	800	17	0.0318	0.83	936	0.000853	Signal	0.01	WT H T 2
0001646_05	800	18	0.0801	0.898	936	0.000874	Signal	0.01	WT H T 3
0001646_05	800	19	0.00311	0.774	936	0.000824	Signal	0.00	WT H T 4
0001646_05	800	20	0.116	0.938	936	0.000878	Signal	0.01	WT H T 5
0001646_05	800	21	0.0718	0.902	936	0.000887	Signal	0.01	WT H T 6
30 kDa obere Bande									
0001646_05	800	22	0.763	1.56	915	0.000868	Signal	0.18	WT H T 0
0001646_05	800	23	0.797	1.62	915	0.000894	Signal	0.22	WT H T 1
0001646_05	800	24	0.879	1.74	915	0.000941	Signal	0.15	WT H T 2
0001646_05	800	25	1.02	1.86	915	0.000918	Signal	0.16	WT H T 3
0001646_05	800	26	0.488	1.27	915	0.00085	Signal	0.08	WT H T 4
0001646_05	800	27	1.27	2.11	915	0.000923	Signal	0.12	WT H T 5
0001646_05	800	28	1.32	2.16	915	0.000917	Signal	0.19	WT H T 6
30 kDa untere Bande									
0001646_05	800	29	0.0651	0.95	915	0.000967	Signal	0.02	WT H T 0
0001646_05	800	30	0.0389	0.998	915	0.00105	Signal	0.01	WT H T 1
0001646_05	800	31	0.017	1.15	915	0.00123	Signal	0.00	WT H T 2
0001646_05	800	32	0.109	1.14	915	0.00112	Signal	0.02	WT H T 3
0001646_05	800	33	0.0647	0.957	915	0.000975	Signal	0.01	WT H T 4
0001646_05	800	34	0.0726	1.29	915	0.00133	Signal	0.01	WT H T 5
0001646_05	800	35	0.0996	1.08	915	0.00108	Signal	0.01	WT H T 6
260 kDa									
0001646_05	800	36	-0.00526	0.722	1083	0.000672	Signal	0.00	WT H T 0
0001646_05	800	37	0.0129	0.734	1083	0.000666	Signal	0.00	WT H T 1
0001646_05	800	38	0.0128	0.725	1083	0.000658	Signal	0.00	WT H T 2
0001646_05	800	39	0.0117	0.723	1083	0.000657	Signal	0.00	WT H T 3
0001646_05	800	40	0.0181	0.749	1083	0.000675	Signal	0.00	WT H T 4
0001646_05	800	41	0.0177	0.77	1083	0.000695	Signal	0.00	WT H T 5
0001646_05	800	42	0.0195	0.761	1083	0.000684	Signal	0.00	WT H T 6

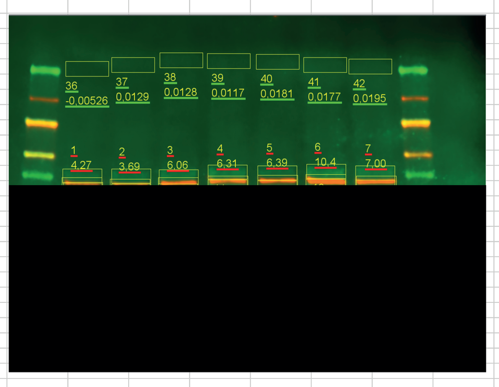

	Human WT						
70 kDa upper band	day 0	day 1	day 2	day 3	day 4	day 5	day 6
	0.016	0.007	0.016	0.016	0.013	0.020	0.021
	0.023	0.029	0.037	0.059	0.008	0.027	0.026
	0.014	0.021	0.018	0.025	0.005	0.015	0.017
	0.006	0.020	0.009	0.022	0.005	0.018	0.008
mean	0.01	0.02	0.02	0.03	0.01	0.02	0.02

70 kD lower band	day 0	day 1	day 2	day 3	day 4	day 5	day 6
	0.008	0.007	0.015	0.019	0.008	0.019	0.023
	0.015	0.013	0.028	0.056	0.010	0.016	0.036
	0.000	0.002	0.005	0.013	0.000	0.011	0.010
	0.004	0.011	0.006	0.023	0.002	0.016	0.008
mean	0.007	0.008	0.013	0.027	0.005	0.016	0.019
	0.024	0.014	0.031	0.035	0.021	0.039	0.044
	0.038	0.042	0.065	0.115	0.018	0.043	0.062
	0.015	0.023	0.024	0.038	0.006	0.027	0.027
	0.010	0.030	0.014	0.045	0.007	0.034	0.016
mean	0.022	0.027	0.033	0.058	0.013	0.035	0.037
	0.013	0.012	0.022	0.038	0.008	0.007	0.020

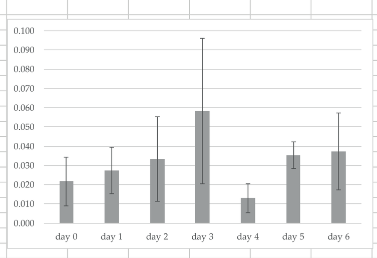

30 kDa obere Bande	day 0	day 1	day 2	day 3	day 4	day 5	day 6
	0.17	0.17	0.11	0.14	0.07	0.12	0.15
	0.02	0.04	0.03	0.06	0.02	0.05	0.06
	0.18	0.22	0.15	0.16	0.08	0.12	0.19
	0.08	0.24	0.07	0.20	0.04	0.14	0.09
mean	0.11	0.17	0.09	0.14	0.05	0.11	0.12
	0.074	0.090	0.050	0.056	0.027	0.041	0.059

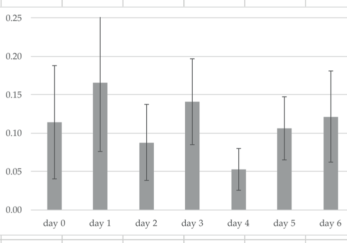

Western Blot #80 Gel 1									
Image Name	Channel	Name	Signal	Total	Area	Bkgnd.	Type		
0001650_05	700	1	5.04	7.36	1044	0.00222	Signal		WT H T 0
0001650_05	700	2	7.02	9.39	1044	0.00227	Signal		WT H T 2
0001650_05	700	3	7.72	10.1	1044	0.00228	Signal		WT H T 4
0001650_05	700	4	8.63	10.8	1044	0.00209	Signal		WT H T 6
70 kDa obere Bande									
0001650_05	800	5	0.0285	0.726	972	0.000717	Signal	0.01	WT H T 0
0001650_05	800	6	0.0598	0.801	972	0.000762	Signal	0.01	WT H T 2
0001650_05	800	7	0.037	0.767	972	0.000751	Signal	0.00	WT H T 4
0001650_05	800	8	0.0723	0.759	972	0.000707	Signal	0.01	WT H T 6
70 kDa untere Bande									
0001650_05	800	9	0.0198	0.713	972	0.000713	Signal	0.00	WT H T 0
0001650_05	800	10	0.0389	0.795	972	0.000778	Signal	0.01	WT H T 2
0001650_05	800	11	0.0189	0.754	972	0.000756	Signal	0.00	WT H T 4
0001650_05	800	12	0.0663	0.812	972	0.000767	Signal	0.01	WT H T 6
30 kDa obere Bande									
0001650_05	800	13	0.424	0.908	684	0.000708	Signal	0.08	WT H T 0
0001650_05	800	14	0.475	1.03	684	0.000812	Signal	0.07	WT H T 2
0001650_05	800	15	0.328	0.872	684	0.000795	Signal	0.04	WT H T 4
0001650_05	800	16	0.778	1.37	684	0.00087	Signal	0.09	WT H T 6
30 kD untere Bande									
0001650_05	800	17	0.0145	0.537	684	0.000764	Signal	0.00	WT H T 0
0001650_05	800	18	0.0169	0.74	684	0.00106	Signal	0.00	WT H T 2
0001650_05	800	19	0.0257	0.699	684	0.000985	Signal	0.00	WT H T 4
0001650_05	800	20	0.0218	0.87	684	0.00124	Signal	0.00	WT H T 6
260 kDa									
0001650_05	800	21	0.00921	0.572	884	0.000637	Signal	0.00	WT H T 0
0001650_05	800	22	-0.00318	0.539	884	0.000614	Signal	0.00	WT H T 2
0001650_05	800	23	-0.00454	0.53	884	0.000604	Signal	0.00	WT H T 4
0001650_05	800	24	0.01	0.559	884	0.000621	Signal	0.00	WT H T 6

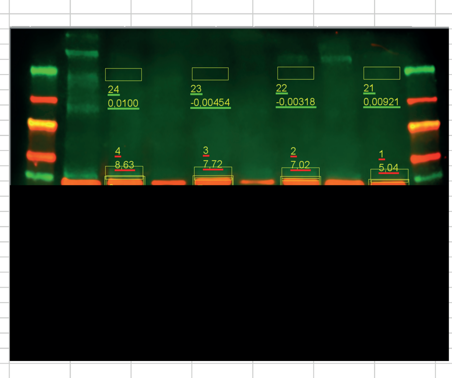

Western Blot #80/2 Gel 1									
Image Name	Channel	Name	Signal	Total	Area	Bkgnd.	Type		
0001655_05	700	1	2.29	3.45	825	0.00141	Signal		WT H T 1
0001655_05	700	2	3.35	4.6	825	0.00151	Signal		WT H T 3
0001655_05	700	3	6.83	8.11	825	0.00156	Signal		WT H T 5
70 kDa obere Bande									
0001655_05	800	4	0.0447	0.303	833	0.00031	Signal	0.02	WT H T 1
0001655_05	800	5	0.0735	0.341	833	0.000321	Signal	0.02	WT H T 3
0001655_05	800	6	0.123	0.376	833	0.000303	Signal	0.02	WT H T 5
70 kDa untere Bande									
0001655_05	800	7	0.0247	0.316	833	0.00035	Signal	0.01	WT H T 1
0001655_05	800	8	0.0768	0.362	833	0.000342	Signal	0.02	WT H T 3
0001655_05	800	9	0.109	0.402	833	0.000351	Signal	0.02	WT H T 5
30 kDa obere Bande									
0001655_05	800	10	0.543	0.88	854	0.000394	Signal	0.24	WT H T 1
0001655_05	800	11	0.656	0.988	854	0.000389	Signal	0.20	WT H T 3
0001655_05	800	12	0.943	1.33	854	0.000456	Signal	0.14	WT H T 5
30 kD untere Bande									
0001655_05	800	13	0.0194	0.399	854	0.000444	Signal	0.01	WT H T 1
0001655_05	800	14	0.00133	0.422	854	0.000493	Signal	0.00	WT H T 3
0001655_05	800	15	0.0228	0.595	854	0.00067	Signal	0.00	WT H T 5
260 kDa									
0001655_05	800	16	0.000268	0.28	1056	0.000265	Signal	0.00	WT H T 1
0001655_05	800	17	0.00416	0.291	1056	0.000271	Signal	0.00	WT H T 3
0001655_05	800	18	0.0134	0.298	1056	0.000269	Signal	0.00	WT H T 5

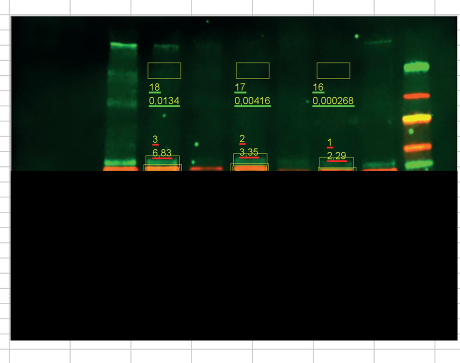

Western Blot #79 Gel 1									
Image Name	Channel	Name	Signal	Total	Area	Bkgnd.	Type		
0001638_04	700	1	2.95	4.87	855	0.00225	Signal		WT H T 0
0001638_04	700	2	2.37	4.54	855	0.00254	Signal		WT H T 1
0001638_04	700	3	6.03	8.35	855	0.00271	Signal		WT H T 2
0001638_04	700	4	5.16	7.12	855	0.00229	Signal		WT H T 3
70 kDa obere Bande									
0001638_04	800	5	0.0479	0.406	770	0.000465	Signal	0.02	WT H T 0
0001638_04	800	6	0.0169	0.5	770	0.000627	Signal	0.01	WT H T 1
0001638_04	800	7	0.0935	0.622	770	0.000686	Signal	0.02	WT H T 2
0001638_04	800	8	0.0847	0.528	770	0.000576	Signal	0.02	WT H T 3
70 kDa untere Bande									
0001638_04	800	9	0.0234	0.401	770	0.000491	Signal	0.01	WT H T 0
0001638_04	800	10	0.0163	0.494	770	0.00062	Signal	0.01	WT H T 1
0001638_04	800	11	0.091	0.658	770	0.000737	Signal	0.02	WT H T 2
0001638_04	800	12	0.0963	0.564	770	0.000608	Signal	0.02	WT H T 3
30 kDa obere Bande									
0001638_04	800	13	0.501	0.989	915	0.000533	Signal	0.17	WT H T 0
0001638_04	800	14	0.414	1.01	915	0.000654	Signal	0.17	WT H T 1
0001638_04	800	15	0.652	1.21	915	0.000612	Signal	0.11	WT H T 2

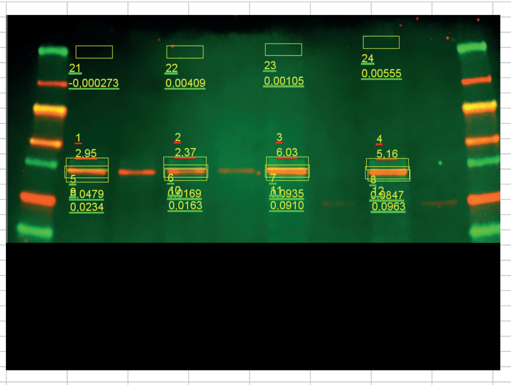

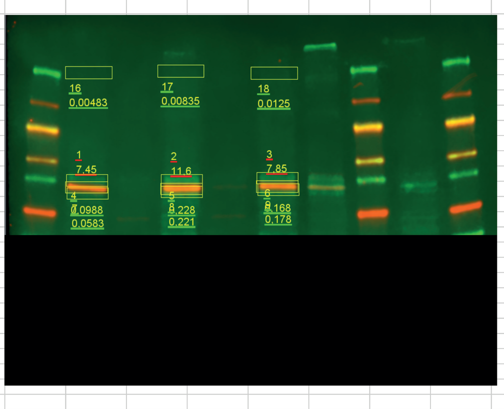

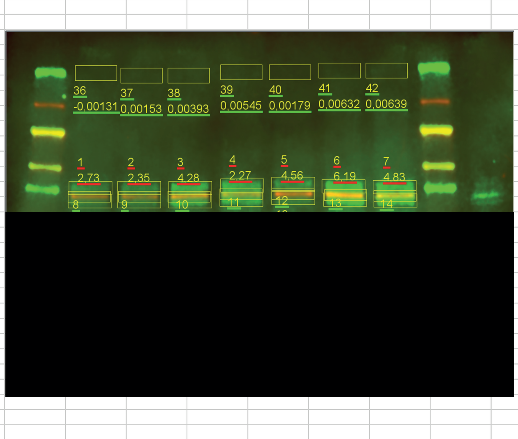

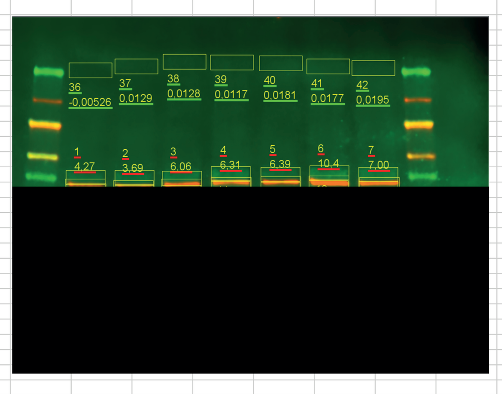

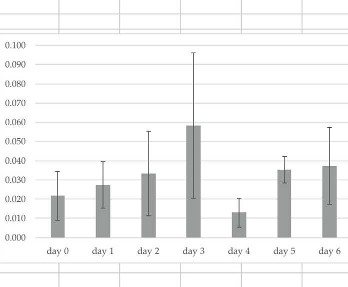

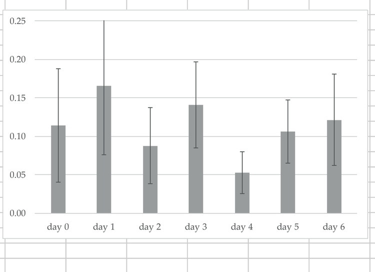

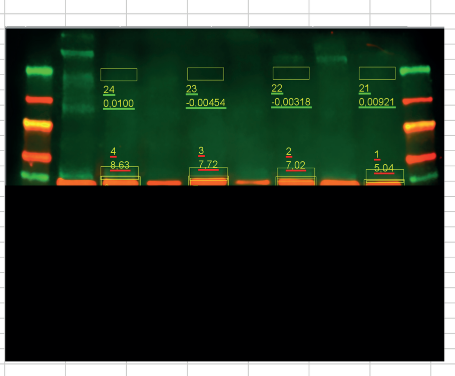

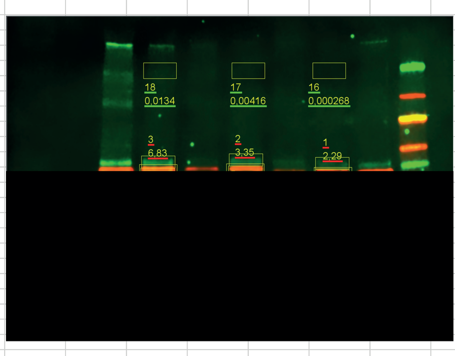

Western Blot #78 Gel 1									
Image Name	Channel	Name	Signal	Total	Area	Bkgnd.	Type		
0001632_04	700	1	5.08	6.39	935	0.00139	Signal		WT M T 1
0001632_04	700	2	11.1	13	935	0.00204	Signal		WT M T 2
0001632_04	700	3	14.6	17.7	935	0.00334	Signal		WT M T 3
0001632_04	700	4	11.8	15.3	935	0.00381	Signal		WT M T 4
0001632_04	700	5	17.2	20.5	935	0.00356	Signal		WT M T 6
70 kDa obere Bande									
0001632_04	800	10	0.151	0.272	588	0.000203	Signal	0.03	WT M T 1
0001632_04	800	11	0.306	0.439	588	0.000226	Signal	0.03	WT M T 2
0001632_04	800	12	0.413	0.549	588	0.000231	Signal	0.03	WT M T 3
0001632_04	800	13	0.133	0.277	588	0.000216	Signal	0.01	WT M T 4
0001632_04	800	14	0.0984	0.232	588	0.000214	Signal	0.01	WT M T 6
30 kDa obere Bande									
0001632_04	800	15	0.0739	0.28	638	0.000281	Signal	0.01	WT M T 1
0001632_04	800	16	0.0882	0.318	638	0.000362	Signal	0.01	WT M T 2
0001632_04	800	17	0.0836	0.353	638	0.000423	Signal	0.01	WT M T 3
0001632_04	800	18	0.182	0.49	638	0.00044	Signal	0.02	WT M T 4
0001632_04	800	19	0.506	0.974	638	0.000734	Signal	0.03	WT M T 6
30 kD untere Bande									
0001632_04	800	20	0.0167	0.214	638	0.000312	Signal	0.00	WT M T 1
0001632_04	800	21	0.00447	0.284	638	0.000438	Signal	0.00	WT M T 2
0001632_04	800	22	0.0000603	0.307	638	0.000476	Signal	0.00	WT M T 3
0001632_04	800	23	0.00589	0.408	638	0.0006	Signal	0.00	WT M T 4
0001632_04	800	24	0.131	0.806	638	0.00123	Signal	0.01	WT M T 6
70 kD untere Bande									
0001632_04	800	30	0.0322	0.167	588	0.000229	Signal	0.01	WT M T 1
0001632_04	800	31	0.0929	0.241	588	0.000261	Signal	0.01	WT M T 2
0001632_04	800	32	0.16	0.337	588	0.000295	Signal	0.01	WT M T 3
0001632_04	800	33	0.0371	0.181	588	0.000224	Signal	0.00	WT M T 4
0001632_04	800	34	0.0556	0.182	588	0.000215	Signal	0.00	WT M T 6

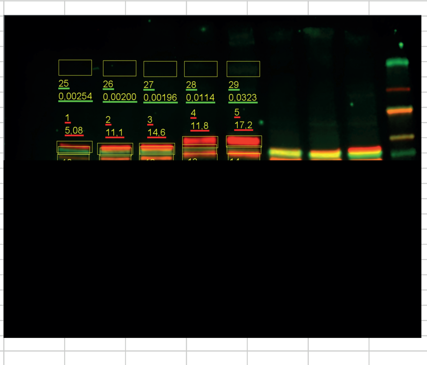

Western Blot #83 Gel 2									
Image Name	Channel	Name	Signal	Total	Area	Bkgnd.	Type		
0001662_05	700	1	10.2	11.5	1120	0.00111	Signal		WT M T 6
0001662_05	700	2	32.2	34.3	1120	0.00182	Signal		WT M T 5
0001662_05	700	3	5.33	6.54	1120	0.00111	Signal		WT M T 4
0001662_05	700	4	8.35	9.61	1120	0.00115	Signal		WT M T 3
0001662_05	700	5	6.68	7.93	1120	0.00116	Signal		WT M T 2
0001662_05	700	6	2.82	4.02	1120	0.00112	Signal		WT M T 1
0001662_05	700	7	6.32	7.55	1120	0.00114	Signal		WT M T 0
70 kDa obere Bande									
0001662_05	800	8	0.0705	0.324	605	0.000333	Signal	0.01	WT M T 6
0001662_05	800	9	0.497	0.789	605	0.000394	Signal	0.02	WT M T 5
0001662_05	800	10	0.08	0.299	605	0.0003	Signal	0.02	WT M T 4
0001662_05	800	11	0.164	0.373	604	0.000282	Signal	0.02	WT M T 3
0001662_05	800	12	0.139	0.353	616	0.000282	Signal	0.02	WT M T 2
0001662_05	800	13	0.101	0.325	605	0.000293	Signal	0.04	WT M T 1
0001662_05	800	14	0.231	0.46	604	0.00031	Signal	0.04	WT M T 0
30 kDa obere Bande									
0001662_05	800	22	1.68	2.45	780	0.000942	Signal	0.16	WT M T 6
0001662_05	800	23	1.22	1.97	782	0.000752	Signal	0.04	WT M T 5
0001662_05	800	24	0.794	1.15	780	0.000414	Signal	0.15	WT M T 4
0001662_05	800	25	0.414	0.72	780	0.000363	Signal	0.05	WT M T 3
0001662_05	800	26	0.427	0.742	780	0.000417	Signal	0.06	WT M T 2
0001662_05	800	27	0.302	0.603	780	0.000351	Signal	0.11	WT M T 1
0001662_05	800	28	1.22	1.55	782	0.000379	Signal	0.19	WT M T 0
30 kD untere Bande									
0001662_05	800	29	1.63	2.44	780	0.00101	Signal	0.16	WT M T 6
0001662_05	800	30	1.32	2.31	782	0.00114	Signal	0.04	WT M T 5
0001662_05	800	31	0.612	1.13	780	0.000575	Signal	0.11	WT M T 4
0001662_05	800	32	0.0728	0.382	780	0.000433	Signal	0.01	WT M T 3
0001662_05	800	33	0.0866	0.401	780	0.000425	Signal	0.01	WT M T 2
0001662_05	800	34	0.0601	0.337	780	0.00037	Signal	0.02	WT M T 1
0001662_05	800	35	0.142	0.484	782	0.000455	Signal	0.02	WT M T 0
70 kD untere Bande									
0001662_05	800	15	0.056	0.262	605	0.000331	Signal	0.01	WT M T 6
0001662_05	800	16	0.187	0.442	605	0.00041	Signal	0.01	WT M T 5
0001662_05	800	17	0.0267	0.215	605	0.000301	Signal	0.01	WT M T 4
0001662_05	800	18	0.0708	0.264	604	0.000312	Signal	0.01	WT M T 3
0001662_05	800	19	0.0377	0.246	605	0.000324	Signal	0.01	WT M T 2
0001662_05	800	20	0.0304	0.226	605	0.000319	Signal	0.01	WT M T 1
0001662_05	800	21	0.09	0.312	604	0.00036	Signal	0.01	WT M T 0

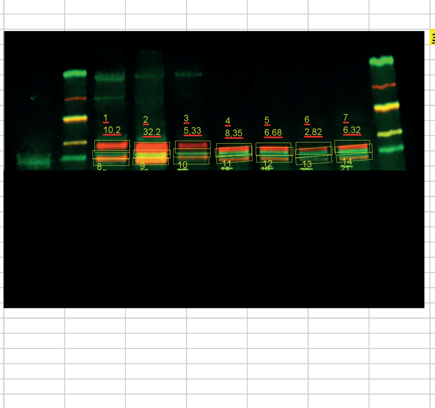

Western Blot #83 Gel 3 11.10.									
Image Name	Channel	Name	Signal	Total	Area	Bkgnd.	Type		
0001665_05	700	1	0.0106	1.46	1210	0.0012	Signal		
0001665_05	700	2	15.7	17.9	1210	0.00184	Signal		
0001665_05	700	3	1.07	2.63	1210	0.00129	Signal		
0001665_05	700	4	3.65	5.16	1210	0.00125	Signal		
0001665_05	700	5	0.288	1.75	1210	0.00121	Signal		
0001665_05	700	6	1.42	2.91	1210	0.00123	Signal		
70 kDa obere Bande									
0001665_05	800	8	0.348	0.728	702	0.000541	Signal	0.02	WT Maus T 5
0001665_05	800	9	0.0408	0.354	702	0.000447	Signal	0.04	WT Maus T 4
0001665_05	800	10	0.102	0.395	702	0.000418	Signal	0.03	WT Maus T 3
0001665_05	800	11	0.0135	0.3	702	0.000408	Signal	0.05	WT Maus T 1
0001665_05	800	12	0.0857	0.384	702	0.000425	Signal	0.06	WT Maus T 0
30 kDa obere Bande									
0001665_05	800	14	0.706	1.47	854	0.000891	Signal	0.04	WT Maus T 5
0001665_05	800	15	0.267	0.777	854	0.000597	Signal	0.25	WT Maus T 4
0001665_05	800	16	0.118	0.52	854	0.000471	Signal	0.03	WT Maus T 3
0001665_05	800	17	0.0534	0.42	854	0.000429	Signal	0.19	WT Maus T 1
0001665_05	800	18	0.676	1.08	854	0.000478	Signal	0.48	WT Maus T 0
30 kD untere Bande									
0001665_05	800	19	1.17	2.3	854	0.00132	Signal	0.07	WT Maus T 5
0001665_05	800	20	0.676	1.28	854	0.000706	Signal	0.63	WT Maus T 4
0001665_05	800	21	0.117	0.56	854	0.000519	Signal	0.03	WT Maus T 3
0001665_05	800	22	0.027	0.424	854	0.000465	Signal	0.09	WT Maus T 1
0001665_05	800	23	0.138	0.62	854	0.000565	Signal	0.10	WT Maus T 0
70 kD untere Bande									
0001665_05	800	31	0.138	0.508	702	0.000527	Signal	0.01	WT Maus T 5
0001665_05	800	32	0.0372	0.352	702	0.000448	Signal	0.03	WT Maus T 4
0001665_05	800	33	0.0427	0.354	702	0.000443	Signal	0.01	WT Maus T 3
0001665_05	800	34	0.00611	0.3	702	0.000419	Signal	0.02	WT Maus T 1
0001665_05	800	35	0.0254	0.36	702	0.000476	Signal	0.02	WT Maus T 0

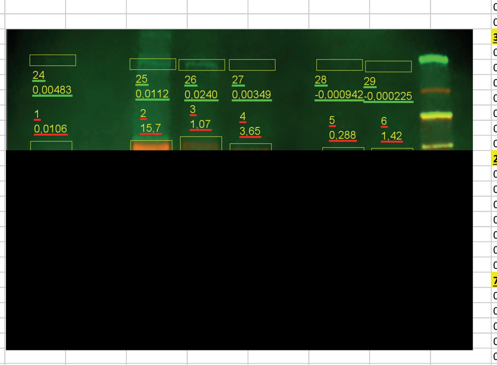

Western Blot #78 Gel 2 8.9.									
Image Name	Channel	Name	Signal	Total	Area	Bkgnd.	Type		
0001631_05	700	1	9.74	11.2	750	0.00189	Signal		
70 kDa obere Bande									
0001631_05	800	2	0.518	0.629	600	0.000184	Signal	0.053	WT M T 2
30 kDa obere Bande									
0001631_05	800	3	0.161	0.401	702	0.000342	Signal	0.017	WT M T 2
30 kD untere Bande									
0001631_05	800	4	0.0787	0.355	728	0.000379	Signal	0.008	WT M T 2
70 kD untere Bande									
0001631_05	800	6	0.158	0.298	588	0.000237	Signal	0.016	WT M T 2

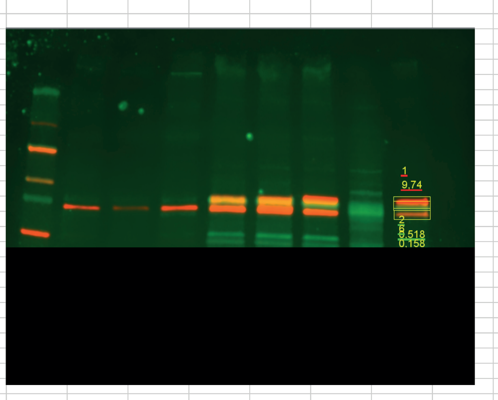

	Maus WT						
70 kDa obere Bande	day 0	day 1	day 2	day 3	day 4	day 5	day 6
			0.05				
		0.03	0.03	0.03	0.01		0.01
	0.04	0.04	0.02	0.02	0.02	0.02	0.01
	0.06	0.05		0.03	0.04	0.02	
	0.07	0.08	0.21	0.04	0.03	0.03	0.01
Median		0.041	0.040	0.028	0.024	0.022	0.007

70 kD untere Bande	day 0	day 1	day 2	day 3	day 4	day 5	day 6
			0.02				
		0.01	0.01	0.01	0.00		0.00
	0.01	0.01	0.01	0.01	0.01	0.01	0.01
	0.02	0.02		0.01	0.03	0.01	
	0.03	0.02	0.07	0.01	0.01	0.01	0.01
Median	0.018	0.016	0.012	0.011	0.009	0.009	0.005
		0.036	0.036	0.039	0.014		0.009
	0.051	0.047	0.026	0.028	0.020	0.021	0.012
	0.078	0.068		0.040	0.073	0.031	0.000
	0.098	0.099		0.054	0.045	0.039	0.021
			0.069				
	0.076	0.062	0.044	0.040	0.038	0.031	0.014
	0.024	0.028	0.023	0.011	0.027	0.009	0.009

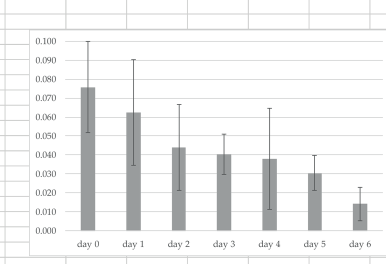

30 kDa obere Bande	day 0	day 1	day 2	day 3	day 4	day 5	day 6
			0.017				
		0.015	0.008	0.006	0.015		0.029
	0.193	0.107	0.064	0.050	0.149	0.038	0.165
	0.476	0.185		0.032	0.250	0.045	
	0.275	0.087	0.137	0.022	0.170	0.036	0.189
Median	0.315	0.099	0.056	0.027	0.146	0.040	0.177
	0.146	0.070	0.059	0.018	0.097	0.005	0.017

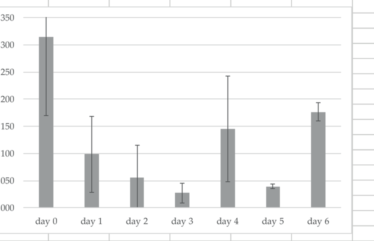

Western Blot #83 Gel 1									
Image Name	Channel	Name	Signal	Total	Area	Bkgnd.	Type		
0001661_06	700	1	6.19	7.68	1134	0.00133	Signal		WT M T 6
0001661_06	700	2	24	26.9	1134	0.00261	Signal		WT M T 5
0001661_06	700	3	2.5	3.99	1134	0.00132	Signal		WT M T 4
0001661_06	700	4	4.83	6.35	1134	0.00134	Signal		WT M T 3
0001661_06	700	5	1.02	2.53	1134	0.00133	Signal		WT M T 2
0001661_06	700	6	1.31	2.69	1134	0.00122	Signal		WT M T 1
0001661_06	700	7	4.65	6.02	1134	0.0012	Signal		WT M T 0
70 kDa obere Bande									
0001661_06	800	8	0.0887	0.37	605	0.000465	Signal	0.01	WT M T 6
0001661_06	800	9	0.737	1.05	605	0.000525	Signal	0.03	WT M T 5
0001661_06	800	10	0.0813	0.349	604	0.000444	Signal	0.03	WT M T 4
0001661_06	800	11	0.198	0.476	605	0.000459	Signal	0.04	WT M T 3
0001661_06	800	12	0.211	0.479	605	0.000443	Signal	0.21	WT M T 2
0001661_06	800	13	0.1	0.353	605	0.000418	Signal	0.08	WT M T 1
0001661_06	800	14	0.334	0.586	604	0.000417	Signal	0.07	WT M T 0
30 kDa obere Bande									
0001661_06	800	15	1.17	1.84	627	0.00107	Signal	0.19	WT M T 6
0001661_06	800	16	0.864	1.63	627	0.00122	Signal	0.04	WT M T 5
0001661_06	800	17	0.425	0.816	626	0.000625	Signal	0.17	WT M T 4
0001661_06	800	18	0.106	0.419	627	0.0005	Signal	0.02	WT M T 3
0001661_06	800	19	0.14	0.451	627	0.000497	Signal	0.14	WT M T 2
0001661_06	800	20	0.114	0.397	627	0.000452	Signal	0.09	WT M T 1
0001661_06	800	21	1.28	1.64	626	0.000572	Signal	0.28	WT M T 0
30 kD untere Bande									
0001661_06	800	22	1.08	2.35	627	0.0015	Signal	0.17	WT M T 6
0001661_06	800	23	1.16	2.28	627	0.00179	Signal	0.05	WT M T 5
0001661_06	800	24	0.553	1.14	627	0.000934	Signal	0.22	WT M T 4
0001661_06	800	25	0.0309	0.389	627	0.000571	Signal	0.01	WT M T 3
0001661_06	800	26	0.0345	0.433	627	0.000636	Signal	0.03	WT M T 2
0001661_06	800	27	0.0673	0.348	627	0.000447	Signal	0.05	WT M T 1
0001661_06	800	28	0.134	0.491	626	0.000571	Signal	0.03	WT M T 0
260 kDa									
0001661_06	800	29	0.129	0.448	882	0.000361	Signal	0.02	WT M T 6
0001661_06	800	30	0.0262	0.368	882	0.000388	Signal	0.00	WT M T 5
0001661_06	800	31	0.0302	0.339	882	0.00035	Signal	0.01	WT M T 4
0001661_06	800	32	0.00266	0.29	882	0.000326	Signal	0.00	WT M T 3
0001661_06	800	33	0.00162	0.294	882	0.000332	Signal	0.00	WT M T 2
0001661_06	800	34	0.000188	0.299	882	0.000338	Signal	0.00	WT M T 1
0001661_06	800	35	0.000731	0.308	882	0.000349	Signal	0.00	WT M T 0
70 kD untere Bande									
0001661_06	800	36	0.0426	0.352	605	0.000512	Signal	0.01	WT M T 6
0001661_06	800	37	0.207	0.552	605	0.000571	Signal	0.01	WT M T 5
0001661_06	800	38	0.0307	0.321	605	0.00048	Signal	0.01	WT M T 4
0001661_06	800	39	0.0645	0.371	605	0.000501	Signal	0.01	WT M T 3
0001661_06	800	40	0.074	0.372	605	0.000493	Signal	0.07	WT M T 2
0001661_06	800	41	0.0299	0.304	605	0.000454	Signal	0.02	WT M T 1
0001661_06	800	42	0.124	0.426	604	0.000499	Signal	0.03	WT M T 0

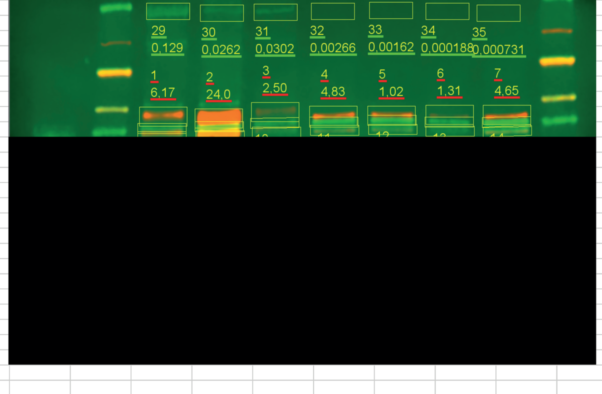

Supplement: Supplementary file 1 [file Supplemental Figure S2.pdf › Supplemental Figure S2.pdf]
